# Synthetic lethality between BRCA1 deficiency and poly(ADP-ribose) polymerase inhibition is modulated by processing of endogenous oxidative DNA damage

**DOI:** 10.1093/nar/gkz624

**Published:** 2019-07-22

**Authors:** Sara Giovannini, Marie-Christine Weller, Simone Repmann, Holger Moch, Josef Jiricny

**Affiliations:** 1 Institute of Molecular Life Sciences of the University of Zurich, Winterthurerstrasse 190, CH-8057 Zurich, Switzerland; 2 Institute of Molecular Cancer Research of the University of Zurich, Winterthurerstrasse 190, CH-8057 Zurich, Switzerland; 3 Institute of Biochemistry of the Swiss Federal Institute of Technology, Otto-Stern-Weg 3, CH-8093 Zurich, Switzerland; 4 Institute of Pathology and Molecular Pathology, University Hospital Zurich, Schmelzbergstrasse 12, CH-8091 Zurich, Switzerland

## Abstract

Poly(ADP-ribose) polymerases (PARPs) facilitate the repair of DNA single-strand breaks (SSBs). When PARPs are inhibited, unrepaired SSBs colliding with replication forks give rise to cytotoxic double-strand breaks. These are normally rescued by homologous recombination (HR), but, in cells with suboptimal HR, PARP inhibition leads to genomic instability and cell death, a phenomenon currently exploited in the therapy of ovarian cancers in *BRCA1/2* mutation carriers. In spite of their promise, resistance to PARP inhibitors (PARPis) has already emerged. In order to identify the possible underlying causes of the resistance, we set out to identify the endogenous source of DNA damage that activates PARPs. We argued that if the toxicity of PARPis is indeed caused by unrepaired SSBs, these breaks must arise spontaneously, because PARPis are used as single agents. We now show that a significant contributor to PARPi toxicity is oxygen metabolism. While BRCA1-depleted or -mutated cells were hypersensitive to the clinically approved PARPi olaparib, its toxicity was significantly attenuated by depletion of OGG1 or MYH DNA glycosylases, as well as by treatment with reactive oxygen species scavengers, growth under hypoxic conditions or chemical OGG1 inhibition. Thus, clinical resistance to PARPi therapy may emerge simply through reduced efficiency of oxidative damage repair.

## INTRODUCTION

The seminal discovery of synthetic lethality between defective homologous recombination (HR) and chemical inhibition of poly(ADP-ribose) polymerases (PARPs) led to the development of clinical PARP inhibitors (PARPis) that represent a significant breakthrough in the therapy of familial breast and ovarian cancers linked to mutations in the *BRCA1/2* genes (1–3). The cascade of events leading to synthetic lethality is widely believed to be triggered by single-strand breaks (SSBs). In healthy cells, SSBs rapidly activate PARPs, which help facilitate break repair (4,5). Unrepaired SSBs that persist until S phase collide with replication forks to give rise to one-ended double-strand breaks (DSBs), but these can be rescued by HR (6,7). Chemically-inhibited PARPs remain bound at the SSBs and inhibit their repair (8–10), which increases the number of toxic DSBs. While normal cells can cope with this increase, DSB accumulation in cells with suboptimal HR, such as those carrying *BRCA1/2* mutations, leads to genomic instability and cell death.

The PARPis olaparib, rucaparib, niraparib, talazoparib or veliparib are currently used in the therapy of HR-deficient cancers (11–13). Their efficacy in the treatment of several different types of cancers is currently being tested in a large number of clinical trials, but their current indication is as fourth line therapy of primarily ovarian cancers in patients with *BRCA1/2* mutations who have responded to platinum treatment. Although the emergence of PARPis represents a major breakthrough in cancer therapy, drug resistance has already emerged. This can have several causes: drug efflux through upregulation of multiple drug resistance pathways, partial restoration of HR through secondary mutations in the *BRCA* loci, or inactivation of non-homologous end-joining (NHEJ) pathways that cause genomic instability in HR-deficient cells through error-prone processing of unrepaired DSBs (14–18). However, many cases of resistance are apparently not linked to the above. In an attempt to identify alternative modes of PARPi resistance, we first wanted to elucidate the underlying cause(s) of PARPi cytotoxicity.

We argued that if PARPi toxicity is indeed linked to immobilization of PARPs on SSBs, the breaks must arise spontaneously, because PARPis are used as single agents, rather than in combination with genotoxic substances. However, there are several sources of SSBs in genomic DNA, ranging from aborted adenylated ligation intermediates or type I topoisomerase adducts, gaps between Okazaki fragments and nicks generated by RNases during excision of ribonucleotides from DNA, to cleaved abasic sites resulting from spontaneous base loss or through the removal of aberrant bases by base excision repair (BER) (19). We wanted to learn whether all SSBs contribute equally to PARPi toxicity, or whether there is a subset of breaks, the repair of which is particularly dependent on PARPs.

In this study, we focussed on SSBs associated with BER. Hydrolysis and oxidation of DNA bases represent the greatest and unavoidable threats to genomic integrity. During BER (4), the aberrant bases are excised by one of several specialized DNA glycosylases (MDB4, SMUG1, TDG and UNG2, MYH (MUTYH), NEIL1/2/3, NTHL1, OGG1/2) to leave behind abasic (apurinic/apyrimidinic, AP) sites, which are subsequently cleaved either by AP-endonucleases (APE1/2) or by the intrinsic lyase activity of the glycosylases. Because oxidation represents the most abundant source of aberrant bases in DNA (20), we asked whether SBBs generated during the processing of oxidative DNA damage contribute to PARPi cytotoxicity.

**Figure 1.**
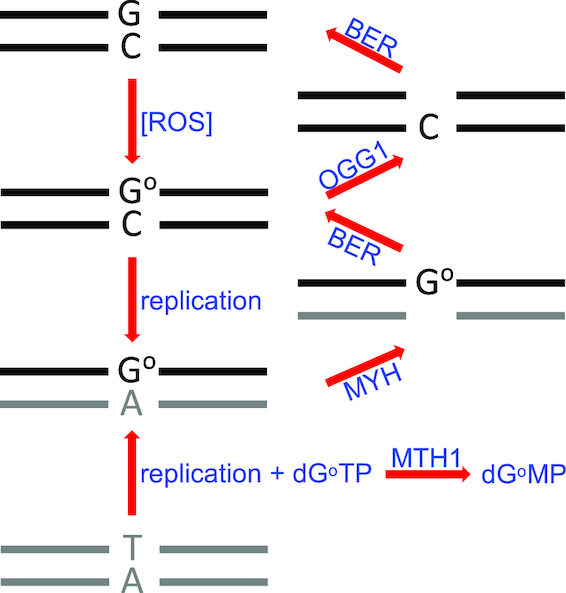
Simplified scheme of G^o^ processing. Oxidation of guanine by ROS gives rise to a G^o^/C base pair. Removal of G^o^ by OGG1, followed by filling of the single nucleotide gap by BER (with the help of pol-β or -λ) restores the original G/C base pair. G^o^ residues remaining in template DNA strand will mispair with A during pol-δ/ϵ catalysed replication. MYH removes the mispaired adenines and BER (with the help of pol-β or -λ) inserts a C opposite the G^o^ to restore a G^o^/C pair, which can be addressed by OGG1/BER to restore the original G/C. If the deoxynucleotide pool is oxidized, dG^o^TP should be hydrolyzed to dG^o^MP by MTH1. Incomplete dG^o^TP hydrolysis can result in G^o^ being inserted opposite template A during replication. The resulting A/G^o^ mispair is addressed by MYH and BER similarly to the G^o^/A mispair.

DNA oxidation gives rise to a large spectrum of aberrant bases [8-oxoguanine, 8-oxoadenine, formamidopyrimidine, thymine glycol, hydroxymethylcytosine and many others (21)] in nuclear and mitochondrial DNA of all organisms (20). The consequences of the presence of these modified or fragmented bases in DNA have been studied in some detail, with early investigations focussing primarily on their mutagenicity. These studies revealed that of all the different structures, G^o^ (8-oxoguanine) was the most deleterious. Indeed, *Escherichia coli* in which the three genes encoding enzymes involved in G^o^ processing (*fpg/mutM, micA/mutY* and *mutT*) have been disrupted display one of the strongest known mutator phenotypes (22). In eukaryotes, G^o^ processing is catalysed primarily by the MutM ortholog OGG1, the MutY homolog MYH (MUTYH) and the MutT homolog MTH1 as indicated in Figure 1. As in *E. coli*, disruption of the three genes encoding these enzymes in mouse resulted in a 37 times higher mutation rate compared to wild-type animals. Importantly, G to T transversions—a hallmark of non-repair of G^o^/A mispairs—represented 96% of mutations (23) and this phenotype is reflected in the tumours of multiple adenomatous polyposis syndrome patients, who carry mutations in *MYH* (24). The latter evidence demonstrates the importance of G^o^ processing by BER in mutagenesis and carcinogenesis, but we wanted to focus on its possible role as a threat to genomic stability in the absence of HR. As mentioned above, removal of aberrant bases by DNA glycosylases results in the generation of abasic sites. These intermediates are highly-reactive and can give rise to e.g. protein/DNA cross-links. This side-reaction is largely avoided by the rapid action of the highly abundant AP-endonucleases, but the SSBs that are generated are not without danger to processes ranging from transcription to replication. It had been postulated that these SSBs are not ‘visible’ to other pathways of DNA metabolism, because BER has been believed to proceed by a concerted, ‘passing the baton’ mechanism, in which the glycosylase hands over to the AP-endonuclease, which then hands over to the polymerase that then passes the final intermediate to the DNA ligase (25). We have shown earlier that this may not always be the case, given that SSBs generated during G^o^ processing are visible to the mismatch repair system (26).

Here, we show that—if unrepaired—SSBs arising during oxidative damage processing are channelled to HR and significantly contribute to the toxicity of PARPis. Thus, while BRCA1-depleted cells were sensitive to the clinically approved PARPi olaparib, knock-down of OGG1 or MYH DNA glycosylases, or chemical inhibition of OGG1, resulted in marked drug resistance. Moreover, the extent of desensitization to olaparib could be augmented by the reactive oxygen species (ROS) scavenger *N*-acetylcysteine (NAC) or by growing the cells in hypoxic conditions. The level of DNA oxidative damage and its metabolism thus need to be added to the growing list of factors (18) affecting the response of cells and tissues to PARPi treatment.

## MATERIALS AND METHODS

### Cell culture and treatments

The A2780 (ECACC 93112519) cell line was generated from a biopsy of an untreated ovarian cancer and was purchased from ECACC. *TP53* and *BRCA1/2* genes are wild-type. The cells were grown in Dulbecco's-modified Eagle's medium (DMEM) (Gibco) supplemented with 5% fetal calf serum (FCS, Gibco). HEK293 cells (ATCC) were grown in DMEM supplemented with 10% FCS. Both media contained also penicillin (100 U/ml, Gibco) and streptomycin (100 μg/ml, Gibco).

SUM149PT cells were kindly provided by Mark O’Connor (Astra Zeneca, Cambridge, UK). This breast cancer cell line is hemizygous for *BRCA1* and the single *BRCA1* allele carries a frameshift (2288delT) mutation. The cells were cultured in Ham's F-12 medium supplemented with 5% heat-inactivated FCS, 10 mM HEPES.KOH pH 8, 1 μg/ml hydrocortisone and 5 μg/ml insulin.

A 20 mM stock solution of olaparib (AZD2281, Ku-0059436; S1060, Selleckchem) was prepared in dimethyl sulfoxide (DMSO) and stored at −80°C. For the clonogenic assays, the solution was diluted in DMEM and added to cells at the indicated final concentrations. For comet assays and immunofluorescence, the cells were treated with 1 μM olaparib for 24 h. The OGG1-ihibitor TH5487 (27) was used at a final concentration of 0.5 μM for clonogenic assay, 1 h before olaparib treatment. NAC powder (Sigma) was dissolved in sterile water. A 500 mM stock solution was added directly to the cell culture medium to yield a final concentration of 2.5 mM (0.625 mM for SUM149PT). The cells were treated first for 24 h, then again for 30 min prior to olaparib treatment.

Hypoxic conditions were induced by growing the cells in 1% O_2_ concentration in a hypoxic chamber (Ruskinn SCI-tive).

### siRNA transfections

The cells were grown to 30–50% confluency and transfected with 40 pmol siRNA oligonucleotides using Lipofectamine RNAiMAX™ (Invitrogen) according to the manufacturer's instructions. The following oligonucleotides were used: siLuciferase (siLuc): ^5′^CGUACGCGGAAUACUUCGA^3′^; siMYH: ^5′^GCUGACAUAUCAAGUAUAU^3′^ (28); siOGG1: ^5′^UCCAAGGUGUGCGACUGCUGCGACA^3′^ (29); siBRCA1: ^5′^ACCAUACAGCUUCAUAAAUAA^3′^; siRAD51: 5′-GAGCUUGACAAACUACUUC-3′; siRNaseH2: ^5′^GGACUUGGAUACUGAUUAU^3′^ (by Microsynth, Balgach, Switzerland).

### Cell survival assays

Cells were seeded in triplicates in 6-well plates at a density of 300–500 cells per well 72 h after siRNA transfection. Twenty-four hours later, the cells were treated with the indicated concentrations of olaparib and left at 37°C. Colony growth was interrupted after 10–14 days. The cells were washed in phosphate-buffered saline (PBS) and incubated with 0.5% Crystal violet in 20% EtOH for 15 min at room temperature (RT). Crystal violet was removed and gently washed away with H_2_O. The colonies were counted when dry. Cell survival after the treatment was shown as percentage of the viability of the untreated cells in a line chart, reporting the average of three independent assays with standard deviation and significance calculated by Two-Way ANOVA (*P*-value < 0.05 *, < 0.01 **, < 0.001 ***, < 0.0001 ****).

### Alkaline comet assays

The CometAssay^®^ kit from Trevigen^®^ was used as described. Seventy-two hours after transfection with siRNA cells were exposed to 1 μM olaparib for 24 h. Cells were re-suspended in ice-cold PBS at a concentration of 3 × 10^5^ cells/ml, embedded in molten low melting temperature agarose at a ratio of 1:10 and spread on CometSlides™. The slides were immersed in 4°C Lysis Solution ON before exposure to Alkaline Unwinding Solution (300 mM NaOH, 1 mM ethylenediaminetetraacetic acid (EDTA), pH>13) for 1 h at 4°C and electrophoresis in chilled Alkaline Electrophoresis Solution (300 mM NaOH, 1 mM EDTA, pH>13) at 21 V for 30 min. Subsequently, the slides were washed twice in distilled H_2_O, immersed in 70% ethanol for 5 min, dried at 37°C and stained with SYBR^®^ Green for 30 min. Images were captured with an Olympus IX81 fluorescence microscope and at least 80 cells were analysed in each of three independent experiments by Image J software.

The analysis of the average of three independent assays with standard deviation and significance calculated by Two-Way ANOVA (*P*-value < 0.05 *, < 0.01 **, < 0.001 ***, < 0.0001 ****) was reported in a bar graph.

### Immunofluorescence

Cells were seeded in 6-well plates (300 000/well) on sterilized coverslips 72 h after siRNA transfection. After adhesion, 1 μM olaparib treatment was performed for 24 h. Fixation for 10 min at RT in 4% formaldehyde followed two washings in PBS. Coverslips were washed twice again for 5 min and the cells were permeabilized in 0.3% Triton/PBS for 5 min at RT. After two more washings, 5% bovine serum albumin (BSA)/PBS blocking was carried out for 1 h at RT. The primary antibody against γ-H2AX (Ser139) (Merk Millipore 05–636, Mouse) was used at a 1:250 dilution in 5% BSA/PBS for 1 h in a humidified chamber. After washing, the coverslips were incubated for 30 min with the secondary antibody AlexaFluor 488 (Invitrogen A11029, goat anti-mouse), diluted 1:100 in 5% BSA/PBS. The coverslips were then washed twice in PBS and once in distilled H_2_O, dried and put on slides with mounting medium containing DAPI. Images were taken with the Olympus IX81 fluorescence microscope. At least 100 cells per condition were analysed. The analysis of the average of three independent assays with standard deviation and significance calculated by Two-Way ANOVA (*P*-value < 0.05 *, < 0.01 **, < 0.001 ***, < 0.0001 ****) was reported in a bar graph.

### Immunohistochemistry

Formalin-fixed, paraffin-embedded tumour sections (2.5 μm) were transferred to glass slides. Immunohistochemistry was carried out using the automated Leica BOND system and the Bond Polymer Refine Detection Kit (Leica Biosystems). G^o^ was visualized using a mouse monoclonal 8-oxoguanine primary antibody (ab64548, Abcam, diluted 1:250). The immunostained slides were scanned using the NanoZoomer Digital Slide Scanner (Hamamatsu Photonics).

## RESULTS

### Knock-down of OGG1 attenuates the sensitivity of BRCA1-depleted cells to olaparib

As mentioned above, removal of modified bases by DNA glycosylases gives rise to abasic (apurinic, apyrimidinic, AP) sites, which are cleaved by AP-endonucleases. That cleaved AP-sites contribute to PARPi toxicity could be shown by the synthetic lethality between PARP inhibition and knock-down of XRCC1 (10,30), a cofactor of DNA ligase III that is involved in the final step of BER. However, because most AP-sites are channelled to the XRCC1/LigIII-dependent step, the above evidence does not provide any information regarding their source. Moreover, XRCC1 is a scaffold protein that has several partners active in different DNA repair pathways (31).

Because G^o^ represents the most abundant aberrant DNA base in both genomic and mitochondrial DNA (32), we postulated that a significant proportion of spontaneous AP-sites is likely to originate from the processing of this aberrant base. The main glycosylase responsible for G^o^ removal is OGG1, which possesses an intrinsic DNA lyase activity. This cleaves the 3′ side of the AP-site (33,34), such that the subsequent cleavage at its 5′ side by the AP-endonuclease generates a single nucleotide gap rather than a nick. We argued that the latter lesions might persist longer than simple nicks and may thus be more readily detected by PARPs. Inhibition or depletion of OGG1 would therefore be expected to reduce the number of SSBs and gaps, and thus decrease the toxicity of PARPis in HR-deficient cells.

**Figure 2.**
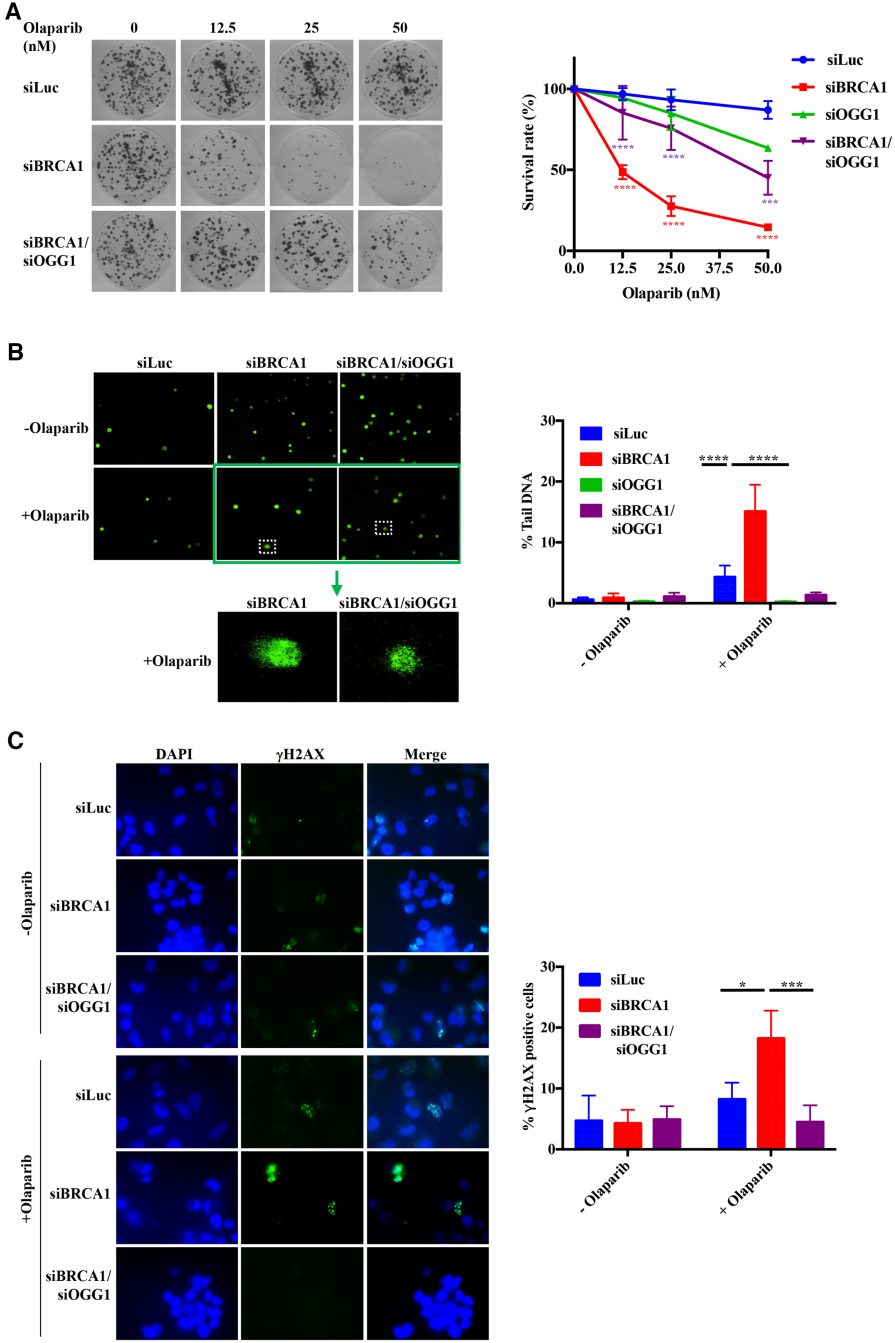
The synthetic lethality of PARP inhibition and BRCA1 deficiency is attenuated by depletion of the 8-oxoguanine DNA glycosylase OGG1. (**A**) The hypersensitivity of BRCA1-depleted human ovarian carcinoma cell line A2780 to olaparib treatment as measured by clonogenic assays is partially rescued by a reduction in the levels of OGG1 (Supplementary Figure S1A and B). The data are normalized to untreated cells and represent a mean of at least three independent experiments, each carried out in triplicate ± s.d.. siLuc, control cells treated with siRNA against luciferase. Asterisks indicate levels of statistical significance, calculated by Two-Way ANOVA test (*P*-value < 0.05 *, < 0.01 **, < 0.001 ***, < 0.0001 ****). Significance: siLuc—siBRCA1; siBRCA1—siBRCA1/siOGG1. (**B**) OGG1 knock-down attenuates the genotoxicity of olaparib treatment measured by alkaline comet assays. The data represent a mean of at least three independent experiments, each carried out in triplicate ± s.d.. siLuc, control cells treated with siRNA against luciferase. Asterisks indicate levels of statistical significance, calculated by Two-Way ANOVA test (*P*-value < 0.05 *, < 0.01 **, < 0.001 ***, < 0.0001 ****). Significance: (siLuc + olaparib) – (siBRCA1 + olaparib); (siBRCA1 + olaparib) – (siBRCA1/siOGG1 + olaparib). (The images for siOGG1 in A and B were indistinguishable from siLuc and are not shown for reasons of space, but the quantification is shown in green in the graphs.) (**C**) DNA damage signalling visualized by γ-H2AX phosphorylation in olaprib-treated BRCA1-depleted cells is diminished by a reduction in OGG1 levels. The graph shows the percentage of cells with more than 10 foci. At least 100 cells were counted in each field. Asterisks indicate levels of statistical significance, calculated by Two-Way ANOVA test (*P*-value < 0.05 *, < 0.01 **, < 0.001 ***, < 0.0001 ****). Significance: (siLuc + olaparib) – (siBRCA1 + olaparib); (siBRCA1 + olaparib) – (siBRCA1/siOGG1 + olaparib).

That OGG1 levels can vary *in vivo* has recently been demonstrated by the identification of a common single nucleotide polymorphism (SNP rs2304277) at the 3′ end of the *OGG1* gene that is associated with a lower expression of the enzyme and that has been linked to an increased ovarian cancer risk in breast cancer patients carrying mutations in *BRCA1* (35). In order to simulate the situation in the BRCA1-deficient ovarian tumours carrying lower OGG1 amounts, we made use primarily of the human ovarian carcinoma cell line A2780 (ECACC 93112519), which was treated with various combinations of siRNAs (Supplementary Figure S1) and subsequently with olaparib. As shown in Figure 2A, knock-down of BRCA1 caused a substantial sensitization of the cells to PARPi treatment in clonogenic assays, but this hypersensitivity was considerably attenuated by a simultaneous knock-down of OGG1, despite the fact that the knock-down of OGG1 alone was slightly toxic to these cells in the clonogenic assays. Similar results were obtained with human embryonic kidney HEK293 cells (Supplementary Figure S2A).

Quantification of strand breaks by alkaline comet assays (Figure 2B) revealed that olaparib treatment increased the percentage of tail DNA ∼3-fold in the control cells treated with siRNA against luciferase (siLuc). Knock-down of BRCA1 caused a ∼13-fold increase in tail DNA percentage, but this amount was reduced almost to levels seen in untreated cells when both BRCA1 and OGG1 were knocked down. This finding was surprising; because cells treated with both OGG1 and BRCA1 siRNAs were more sensitive to olaparib treatment than the control siLuc cells, but less so than cells treated with BRCA1 siRNA alone, we expected the percentage of tail DNA to reflect the results of the clonogenic assays. This was clearly not the case and, although the result could be explained by the difference in exposure times (continuous versus 24 h) in the two assays, we decided to confirm it by studying the formation of DSBs in the same cells. As shown in Figure 2C, quantification of γ-H2AX foci by indirect immunofluorescence reflected the results of the comet assays, with ∼20% of olaparib-treated BRCA1-depleted cells displaying more than 10 γ-H2AX foci and BRCA1/OGG1 doubly depleted cells showing levels close to untreated background. This suggested that the majority of DNA breaks bound by PARPs in asynchronous cell populations arises through OGG1-catalysed processing of G^o^.

### MYH knock-down also causes partial rescue of olaparib sensitivity in BRCA1-depleted cells

If G^o^ persists in DNA until replication, polymerases δ or ϵ can insert both C and A opposite (36–38). While OGG1 removes G° from G^o^/C pairs (33,34), it does not address G^o^/A mispairs. This is accomplished by the MutY homolog (MYH or MUTYH), which initiates BER by excising the A from the G^o^/A mispair (39). Because BER uses polymerase-β or -λ, which insert preferentially a C opposite the G^o^ in the template strand (40), the repair process restores G^o^/C pairs that can then be re-addressed by OGG1 (Figure 1). However, in contrast to OGG1, MYH is a monofunctional DNA glycosylase that lacks lyase activity (41). The breaks generated by AP-endonuclease during MYH-initiated BER of G^o^/A mispairs arising during DNA replication either through the incorporation of A opposite unrepaired G^o^ in the template strand or, alternatively, by incorporation of G^o^ opposite a template A are thus simple nicks, rather than single nucleotide gaps. We therefore wanted to learn whether these SSBs were also addressed by PARPs and whether MYH depletion also attenuated the sensitivity of the BRCA1 deficient cells to olaparib.

**Figure 3.**
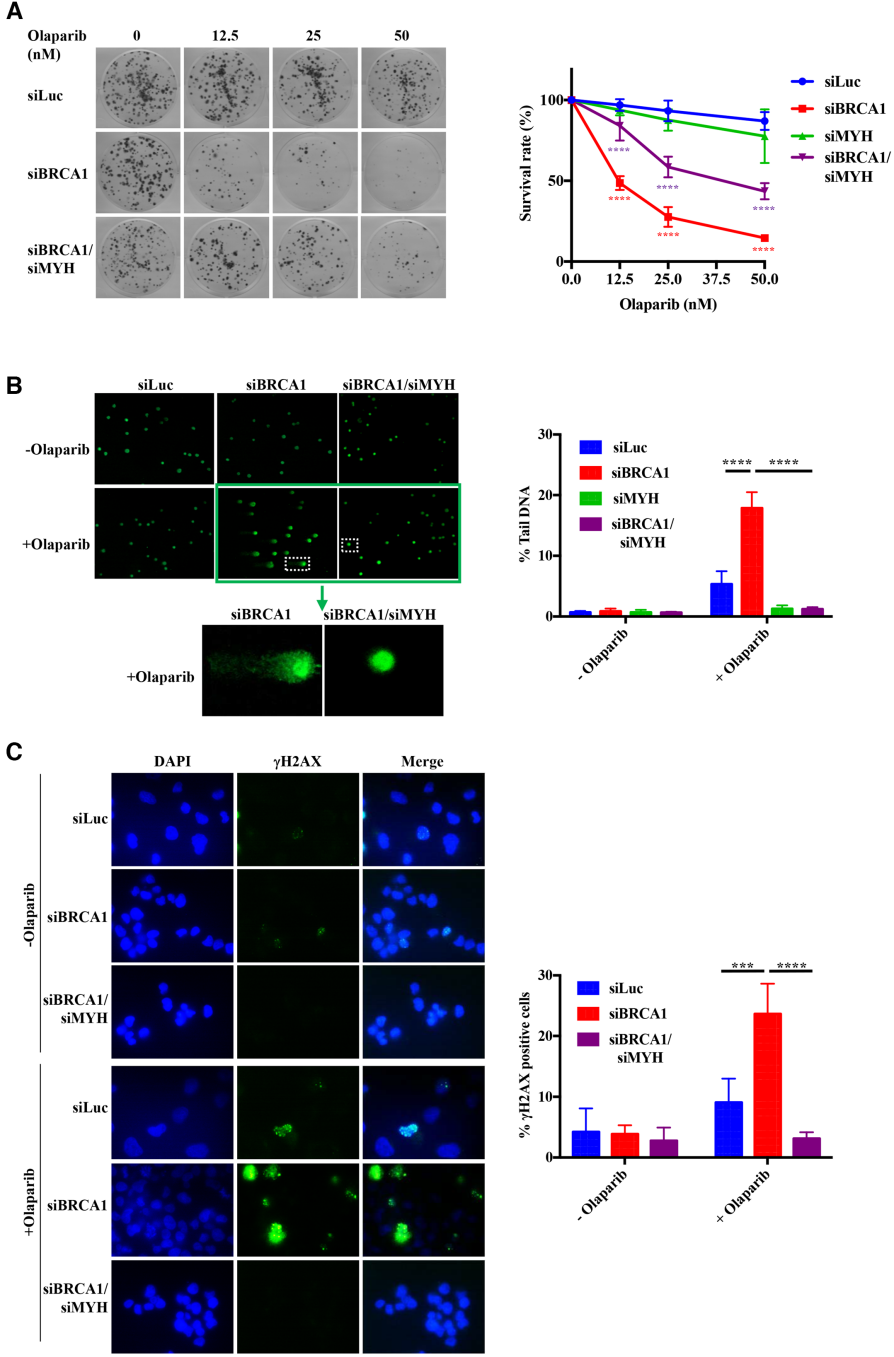
The synthetic lethality of PARP inhibition and BRCA1 deficiency is attenuated by depletion of G^o^/A mismatch-specific adenine glycosylase MYH. (**A**) The hypersensitivity of BRCA1-depleted human ovarian carcinoma cell line A2780 to olaparib treatment as measured by clonogenic assays is partially rescued by a reduction in the levels of MYH (Supplementary Figure S1A and B). The data are normalized to untreated cells and represent a mean of at least three independent experiments, each carried out in triplicate ± s.d.. siLuc, control cells treated with siRNA against luciferase. Asterisks indicate levels of statistical significance, calculated by Two-Way ANOVA test (*P*-value < 0.05 *, < 0.01 **, < 0.001 ***, < 0.0001 ****). Significance: siLuc—siBRCA1; siBRCA1—siBRCA1/siMYH. (**B**) MYH knock-down attenuates the genotoxicity of olaparib treatment measured by alkaline comet assays. The data represent a mean of at least three independent experiments, each carried out in triplicate ± s.d.. siLuc, control cells treated with siRNA against luciferase. Asterisks indicate levels of statistical significance, calculated by Two-Way ANOVA test (*P*-value < 0.05 *, < 0.01 **, < 0.001 ***, < 0.0001 ****). Significance: (siLuc + olaparib) – (siBRCA1 + olaparib); (siBRCA1 + olaparib) – (siBRCA1/siMYH + olaparib). (The images for siMYH in A and B were indistinguishable from siLuc and are not shown for reasons of space, but the quantification is shown in green in the graphs.) (**C**) DNA damage signalling visualized by γ-H2AX phosphorylation in olaprib-treated BRCA1-depleted cells is diminished by a reduction in MYH levels. The graph shows the percentage of cells with more than 10 foci. At least 100 cells were counted in each field. Asterisks indicate levels of statistical significance, calculated by Two-Way ANOVA test (*P*-value < 0.05 *, < 0.01 **, < 0.001 ***, < 0.0001 ****). Significance: (siLuc + olaparib) – (siBRCA1 + olaparib); (siBRCA1 + olaparib) – (siBRCA1/siMYH + olaparib).

The effect of MYH knock-down (Supplementary Figure S1) on cell killing (Figure 3A) and the number of DNA breaks as measured by the comet assay (Figure 3B) or the number of γ-H2AX foci (Figure 3C) were comparable to those seen with OGG1 downregulation. An analogous rescue of cell sensitivity was observed in human embryonic kidney HEK293 cells (Supplementary Figure S2B). This shows that the simple nicks generated by the action of MYH and AP-endonuclease during the processing of G^o^/A mispairs are substrates for PARPs, similarly to the single nucleotide gaps generated by OGG1 and AP-endonuclease during the processing of G^o^/C mispairs.

In order to ensure that the above-described phenomena were not caused by some unspecific effect of BER attenuation with OGG1- or MYH siRNAs, we knocked-down RNaseH2A (Fig S3A). RNaseH2 is responsible for incising DNA at the 5′ side of ribonucleotides misincorporated into DNA during replication (42). In the absence of RNaseH2, topoisomearse I incises DNA at the ribose residue to give rise to PARP-trapping breaks that are highly recombinogenic (43) and that have recently been shown to sensitize RNaseH2-deficient cells to olaparib (44). As shown by pulsed-field gel electrophoresis (PFGE, Supplementary Figure S3B and C), RNaseH2A knock-down gave rise to a similar amount of DSBs as a knock-down of BRCA1, and the amount of breaks in DNA of cells in which both proteins were depleted was additive. As in the comet assays (Figure 3B), the DSB amount in cells doubly-depleted of BRCA1 and MYH was lower than in the siLuc-treated cells (Supplementary Figure S3B and C). This suggests that the effects described above are indeed specific to the depletion of the two oxidation damage-specific enzymes.

### Olaparib toxicity in BRCA1-depleted cells is attenuated by treatments with a ROS scavenger, hypoxia or OGG1 inhibitor

**Figure 4.**
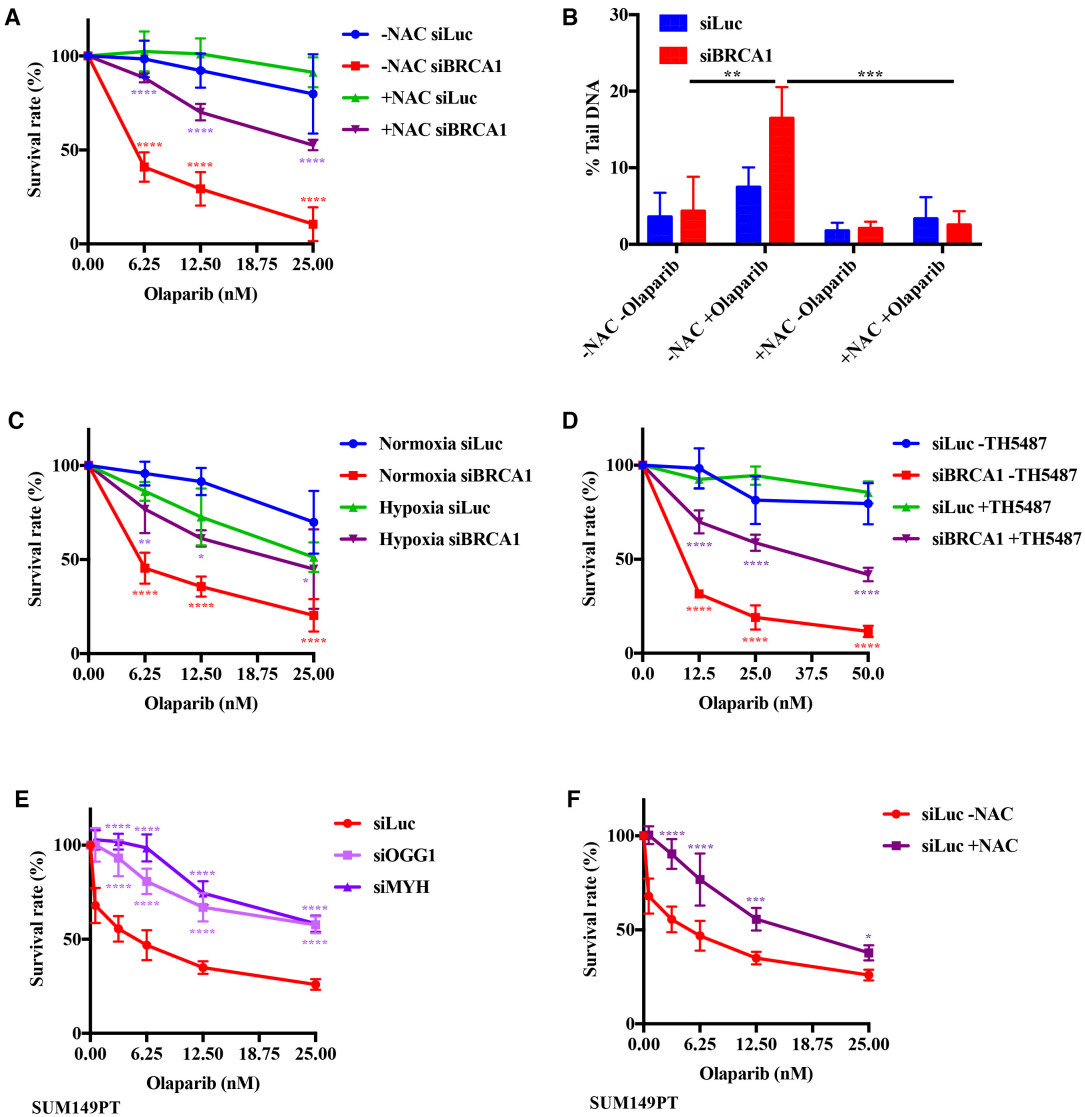
The synthetic lethality of PARP inhibition and BRCA1 deficiency is attenuated by the antioxidant NAC, by hypoxia or by OGG1 inhibition. (**A**) The hypersensitivity of BRCA1-depleted human ovarian carcinoma cell line A2780 to olaparib as measured by clonogenic assays is partially rescued by a treatment with the antioxidant NAC. Significance: (−NAC) siLuc - (−NAC) siBRCA1; (−NAC) siBRCA1 - (+NAC) siBRCA1. (**B**) NAC treatment attenuates the genotoxicity of olaparib in BRCA1-depleted cells measured by alkaline comet assays. Significance: (−NAC, −olaparib) siBRCA1 - (−NAC, + olaparib) siBRCA1; (−NAC, + olaparib) siBRCA1 - (+NAC, +olaparib) siBRCA1. (**C**) BRCA1-depleted human ovarian carcinoma cell line A2780 grown in hypoxic environment (Supplementary Figure S4A) is desensitized to olaparib, as measured by clonogenic assays. Significance: Normox siLuc - Normox siBRCA1; Normox siBRCA1 - Hypox siBRCA1; (**D**) The hypersensitivity of BRCA1-depleted human ovarian carcinoma cell line A2780 to olaparib as measured by clonogenic assays is partially rescued by a treatment with the OGG1 inhibitor TH5487. Significance: siLuc (−TH5487) – siBRCA1 (−TH5487); siBRCA1 (−TH5487) – siBRCA1 (+TH5487). (**E**) The hypersensitivity of triple-negative BRCA1-mutated SUM149PT cells to olaparib as measured by clonogenic assays is partially rescued by pre-treatment with OGG1- or MYH siRNAs (Supplementary Figure S4B). Significance: siLuc – siOGG1; siLuc – siMYH. (**F**) The hypersensitivity of triple-negative BRCA1-mutated SUM149PT cells to olaparib as measured by clonogenic assays is partially rescued by pre-treatment with NAC. Significance: (−NAC) siLuc – (+NAC) siLuc. The results in A–F are means of at least three independent experiments, each carried out in triplicate ± s.d.. Asterisks indicate levels of statistical significance, calculated by Two-Way ANOVA test (*P*-value < 0.05 *, < 0.01 **, < 0.001 ***, < 0.0001 ****).

We wished to confirm the above phenomena further, without having to resort to the use of OGG1 or MYH siRNAs. In order to demonstrate that the partial rescue of olaparib toxicity in BRCA1-depleted cells was indeed linked to the processing of oxidative DNA damage, we studied the olaparib sensitivity in cells treated with the free radical scavenger NAC. Similarly to the downregulation of OGG1 and MYH, NAC treatment also brought about a reduction in the toxicity of the PARPi in the BRCA1 depleted A2780 cells (Figure 4A) and reduced the number of DNA breaks as determined by comet assays (Figure 4B). A similar result was obtained with BRCA1-depleted cells grown under hypoxic (1% oxygen) conditions (Figure 4C and Supplementary Figure S4A), as well as by pre-incubation with the recently described (27) OGG1 inhibitor TH5487 (Figure 4D). That the cells were indeed grown under hypoxic conditions is shown by activation of HIF1α in Supplementary Figure S4A.

### Oxygen metabolism modulates the response of *BRCA1*-mutated breast cancer cells to olaparib

To address the possibility that the above-described phenomena were a peculiarity of the A2780 ovarian cell line or of the siRNA treatments, we repeated the OGG1- and MYH knock-downs (Supplementary Figure S4B) and NAC treatment with the human, *BRCA1*-mutated breast cancer cell line SUM149PT. These cells possess only a single *BRCA1* allele, which carries a frameshift (2288delT) mutation. The outcome of these experiments was very similar to those obtained with the A2780 cells: the toxicity of olaparib was substantially reduced when either glycosylase was depleted (Figure 4E), or when the cells were treated with NAC (Figure 4F).

### Oxygen metabolism modulates also the response of RAD51-depleted cells to olaparib

**Figure 5.**
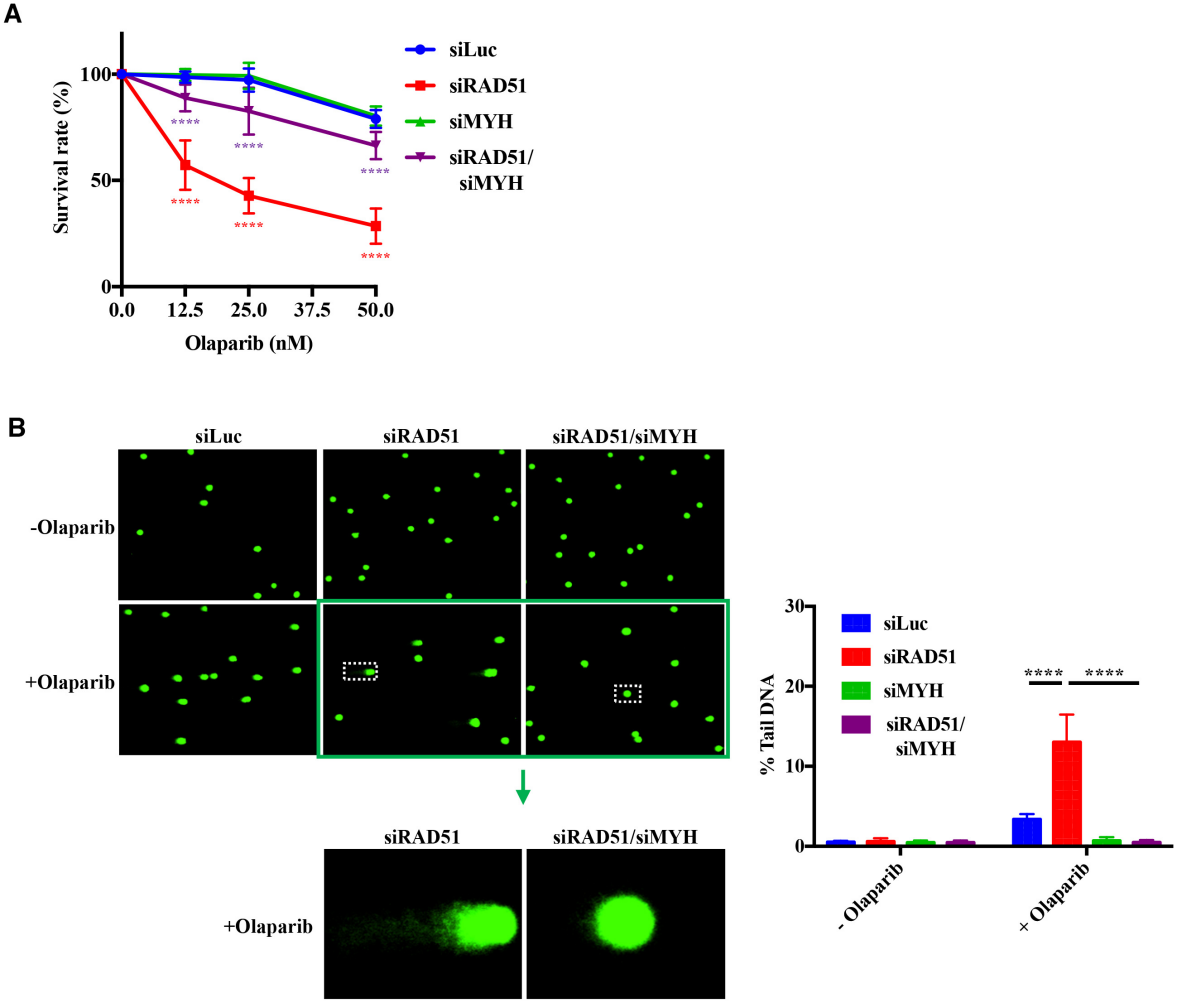
Depletion of MYH glycosylase attenuates the sensitivity of RAD51-depleted cells to olaparib treatment. (**A**) RAD51 and/or MYH were knocked down in the ovarian cancer cell line A2780 with siRNAs (Supplementary Figure S4C) and the sensitivity of the cells to olaparib treatment was assayed by clonogenic assays. Significance: siLuc – siRAD51; siRAD51 – siRAD51/siMYH. (**B**) Accumulation of DNA breaks in the olaparib-treated RAD51- and/or MYH-depleted cells was estimated by comet assays. Significance: (siLuc + olaparib) – (siRAD51 + olaparib); (siRAD51 + olaparib) – (siRAD51/siMYH + olaparib). (The images for siMYH were indistinguishable from siLuc and are not shown for reasons of space, but the quantification is shown in the green columns.) The results in A and B are means of at least three independent experiments, each in triplicate ± s.d.. Asterisks indicate levels of statistical significance, calculated by Two-Way ANOVA test (*P*-value < 0.05 *, < 0.01 **, < 0.001 ***, < 0.0001 ****).

We asked whether the observations described above were limited to BRCA1-depleted cells, or whether oxygen metabolism affected the response to olaparib generally in cells with HR deficiency. We therefore treated the A2780 cells with siRNAs targetting MYH and/or RAD51 (Supplementary Figure S4C). Cells depleted of RAD51, a key member of the HR pathway, were highly sensitive to olaparib as determined by clonogenic assays, whereas MYH-depleted cells were similarly sensitive to the control, siLuc-treated cells. However, as in the case of BRCA1/MYH knock-downs, a combined depletion of MYH and RAD51 desensitized the cells to the PARPi (Figure 5A). The desensitization was reflected in a decrease in the number of DNA breaks as measured by comet assays (Figure 5B).

Taken together, the above data demonstrate that oxygen metabolism is a significant contributor to PARPi cytotoxicity in cells with HR malfunction.

## DISCUSSION

PARP inhibitors represent the first class of cancer chemotherapeutics that target a genetic defect present solely in the tumour cells. They therefore have a very wide therapeutic window and the fact that they are generally well-tolerated helped them gain a rapid approval for clinical use. Most recently, Federal Drug Administration has approved olaparib (Lynparza™) for use as maintenance treatment of adult patients with deleterious or suspected deleterious germline or somatic *BRCA*-mutated advanced epithelial ovarian-, fallopian tube- or primary peritoneal cancer, who showed complete or partial response to first-line platinum-based chemotherapy. However, PARPis might be effective in the treatment of other tumour types, alone or in combination with other therapies, and in order to be able to suggest which tumour types might be sensitive to PARPi therapy, it is important to know how this class of drugs works at the molecular level. Moreover, it is important to understand the reasons underlying the emerging threat of therapy resistance, which has to date been linked to the upregulation of drug efflux pathways, to reactivation of BRCA1/2 function through secondary genetic alterations, inactivation of NHEJ or shieldin, activation of alternative NHEJ pathways, or inactivation of poly-ADP-ribose glycohydrolase (PARG), an enzyme that degrades PAR chains (18). In this work, we uncovered another way to PARPi resistance in BRCA1-depleted cells: a reduction in the efficiency of oxidative damage repair. We show that partial siRNA-mediated knock-down of mRNAs encoding the DNA glycosylases OGG1 (Figure 2) or MYH (Figure 3) is sufficient to desensitize BRCA1-depleted cells to olaparib treatment. We further demonstrate that this effect is not an artefact of siRNA treatments, as similar effects were seen upon chemical inhibition of OGG1 or by growing the cells in the presence of antioxidants or in a hypoxic environment (Figure 4). We also show that the effect is not restricted to BRCA1-depleted cells, because knock-down of RAD51 has similar consequences (Figure 5). We therefore posit that, under normal circumstances, SSBs associated with G^o^ processing are rapidly chaperoned to the downstream steps of BER, possibly through a direct interaction of OGG1 and PARP1 (45). If unrepaired, these breaks are channelled to HR. In cells lacking the latter repair pathway, they become genotoxic through aberrant processing. When the number of unrepaired breaks, such as those bound by inhibited PARP1, is reduced, the cyctotoxicity of PARPis is similarly attenuated. As shown in this study, SSBs generated during G^o^ processing represent a significant proportion of these cytotoxic breaks.

It has ben suggested that there exist two distict pathways of BER: one (including oxidative damage repair) that is PARP1-dependent and the other (including hydrolytic damage repair) that may not require PARP1 (46). It could therefore be anticipated that only the subset of SSBs generated by the PARP1-dependent pathway will contribute to PARPi toxicity. However, our earlier findings demonstrated that SSBs generated not only during G^o^ processing (26), but also those arising during uracil processing, a BER pathway postulated to be PARP1-independent, can be seen by mismatch repair (47). We would therefore argue that all BER-associated SSBs can potentially be channelled to HR and thus play a major role in the toxicity of PARPi in HR-deficient cells.

**Figure 6.**
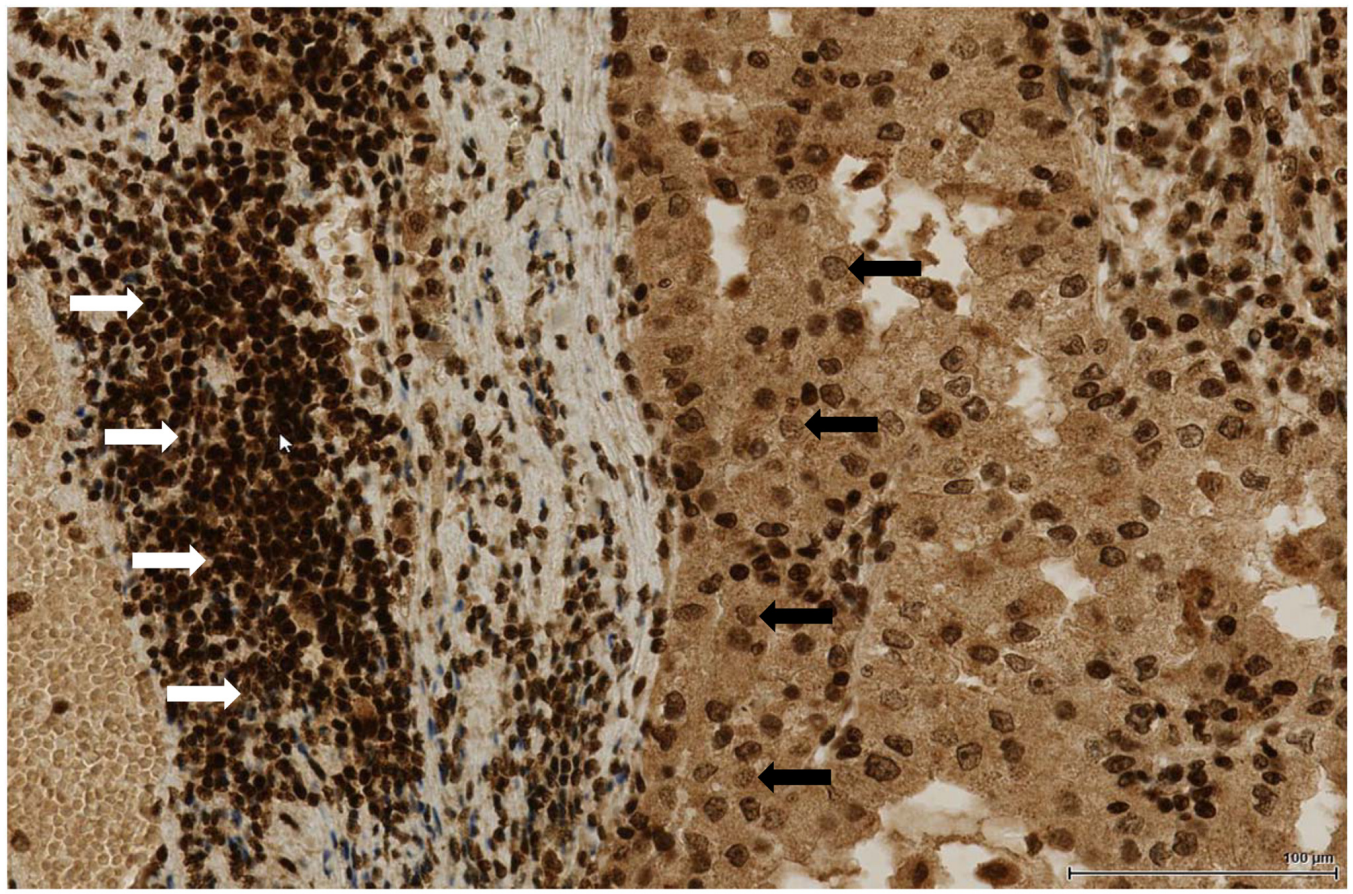
Heterogeneity of G^o^ staining in tumour tissue. Clear cell renal cell carcinoma immunostained using a monoclonal antibody against G^o^. The heavy staining apparent in the peripheral tertiary lymphoid structure containing lymphocytes (white arrows) contrasts with the substantially weaker staining in the tumour cells (black arrows).

Our findings are of substantial relevance to the success of therapy of HR-deficient cancers. They demonstrate that the decreased efficiency of BER can negatively affect the outcome of PARPi therapy. Importantly, this reduction in BER efficiency does not have to be caused by inactivating gene mutations; a mere downregulation of expression of *OGG1* and *MYH* genes, polymorphisms that affect mRNA or protein stability, the interaction of the respective enzymes with their cognate partners, or simply a ROS-poor environment, are all sufficient to make the cells less sensitive to olaparib treatment. This knowledge should make it possible to predict the efficacy of PARPi treatment by an analysis of these parameters in patient DNA or in tumour biopsies. For example, the common polymorphism in *OGG1* rs2304277 has been linked to BRCA1-deficiency in ovarian cancer. The authors of the genetic study (35) anticipated that a decreased amount of OGG1 in the BRCA1-deficient tumour is likely to sensitize it to PARPi therapy. We show that the opposite is true—by not excising G° from DNA, the aberrant base remains and, during replication, can mispair with A to give rise to G to T transversion mutations characteristic of oxidative DNA damage, possibly by exceeding the repair capacity of MYH. In the long run, this mutagenesis is likely to spur tumour progression and transformation to malignancy, but its short term outcome would be resistance to the most promising treatment offered to patients with HR-deficient tumours. Unfortunately, the therapeutic value of the above biomarkers may be limited by tumour heterogeneity. Immunohistochemical ananlysis of G^o^ levels in DNA revealed substantial differences. In the tissue section shown in Figure 6, tumour-infiltrating lymphocytes and tertiary lymphatic structures were very strongly stained. In contrast, tumour cell nuclei were frequently stained rather weakly. It is thus possible that these cells might survive olaparib treatment.

As discussed above, PARPis are currently used in the clinic as single agents, but several attempts have been made to improve their efficacy and overcome drug resistance by combining their use with another therapeutic drug. A recent report demonstrated that PARPi sensitivity is augmented by the depletion of RNaseH2 (44), which removes ribonucleotides from genomic DNA (42). Ribonucleotides that remain in DNA form deleterious adducts with topoisomerase I, and processing of these adducts gives rise to DNA breaks that cause chromosomal rearrangements and that are highly-toxic to HR-deficient cells (43). There have also been attempts to combine PARPis with radiation therapy (48,49). In this scenario, ROS generated by ionizing radiation will cause an increase in the number of DNA breaks and thus also in the sensitivity of the HR-deficient tumour cells to PARPis. Unfortunately, our data predict that the efficacy of PARPi therapy in poorly-oxygenated HR-deficient tumours or tumour regions would be diminished due to the reduced load of oxidative damage and this effect would be important also during standard radiotherapy, to which hypoxic tumours are particularly resistant (50). However, because protons or carbon ions cause water radiolysis, combined therapy with these sources and PARPis might offer a way of overcoming this setback.

It is clear that response to PARPis is affected by many distinct factors. If we are to achieve better therapeutic efficacy, we need to understand not only which factors are involved, but also their interplay and relative importance.

## Supplementary Material

gkz624_Supplemental_File

## Notes

Present address: Marie-Christine Weller, University Hospital Zurich, Department of Oncology and Hematology, Rämistrasse 100, 8091 Zurich, Switzerland.

Present address: Simone Repmann, AbbVie AG, Neuhofstrasse 23, 6341 Baar, Switzerland.
