# Supplementary material for: Synthetic lethality between BRCA1 deficiency and poly(ADP-ribose) polymerase inhibition is modulated by processing of endogenous oxidative DNA damage
Source: Nucleic Acids Res. 2019 Jul 22;47(17):9132–43. doi: 10.1093/nar/gkz624 (PMC6753488; doi:10.1093/nar/gkz624)
Supplement: gkz624_Supplemental_File [file gkz624_supplemental_file.pdf]

SUPPLEMENTARY METHODS

RNA extraction, cDNA, qPCR

RNA was isolated from cells using RNeasy Mini Kit (Qiagen). High Capacity cDNA Reverse Transcription Kit (Applied Biosystems) was used for complementary DNA (cDNA) synthesis. Quantitative real-time SYBR-Green-based PCR reactions were performed with the Light Cycler 480 (Roche). The following primers were used: BRCA1 Fw $^{5'}$ACACAGGTTTGGAGTATGCAAACAGC$^{3'}$ and Rv $^{5'}$TGCAAGGAAGGATTTTCGGGTTCAC$^{3'}$; OGG1 Fw $^{5'}$ACAATCTTTCCGGTGGAGGGAG$^{3'}$ and Rv $^{5'}$CAGGGTAACATCTAGCTGGAAG$^{3'}$; MYH Fw $^{5'}$CATATGCTGTGTGGGTCTCAG$^{3'}$ and Rv $^{5'}$ATTCACCTCCTCCAGGGAAG$^{3'}$; GAPDH Fw $^{5'}$CCCCGGTTTCTATAAATTGAGC$^{3'}$ and Rv $^{5'}$CACCTTCCCCATGGTGTCT$^{3'}$ (Microsynth, Balgach, Switzerland).

Protein extractions and Western blots

The cells were seeded in 10 cm cell culture dishes and treated as indicated. Once the cells were harvested using trypsin, the pellets were washed in cold PBS and quickly frozen in liquid nitrogen. Cells grown under hypoxic conditions (as well as the normoxic controls), were collected by scraping the cells on ice in the hypoxic chamber after 15’ incubation in cold pre-extraction buffer (25 mM Hepes, pH 7.4, 50 mM NaCl, 1 mM EDTA, 3 mM $MgCl_2$, 300 mM sucrose, 0.5% Triton-X-100) supplemented with the proteasome inhibitor MG132, protease and phosphatase inhibitors. After centrifugation and removal of the cytoplasmic fraction, the chromatin pellets were re-suspended in cold SDS lysis buffer (1% SDS, 10 mM Tris HCl, pH 8) supplemented with protease and phosphatase inhibitors and sonicated (20 seconds, 50 cycles, 70% amplitude, Bandelin Sonoplus).
Whole cell extracts for Western blot analysis were prepared by re-suspending the pellets in cold SDS lysis buffer (1% SDS, 10 mM TrisHCl, pH 8) supplemented with protease and phosphatase inhibitors. Sonication was performed with a Bandelin Sonoplus homogenizer for 20 seconds (50 cycles, 70% amplitude).
Protein concentration was determined by the Lowry assay: 3 µl samples were diluted in 97 µl H_2O and incubated for 10 minutes with 1 ml of a 50:1 mixture of Solution A (2% Na_2CO_3 in 0.1 N NaOH) and Solution B (0.5% $CuSO_4 \cdot 5H_2O$ in 1% sodium citrate). 100 µl of a Folin&Ciocalteau's phenol reagent (Sigma) diluted (1:2) in H_2O were added and the mixture was incubated for 30 minutes at RT. Absorbance was measured at 750 nm (Varian-Cary 50 Scan spectrophotometer) and protein concentration was calculated according to a BSA standard curve.
30 µg protein were resuspended in Loading Buffer (5x, 0.25 M Tris pH 6.8, 50% glycerol, 8% SDS, 0.5 mM DTT, 0.1% bromphenol blue), incubated for 5 minutes at 95°C and separated on 4–15% precast polyacrylamide gels (BioRad), or according to size, 6% polyacrylamide gels, using the Mini Trans-Blot Electrophoretic Transfer Cell (BioRad) in 10% SDS-buffer at 60 V. Proteins were transferred in Transfer Buffer (25 mM Tris, 192 mM glycine, 20% methanol) overnight at 4°C at 30 V to a Hybond-P polyvinylidene fluoride (PVDF) membrane (Amersham Pharmacia Biotech) that was previously activated in 100% methanol. Membranes were blocked in 5% non-fat dry milk or BSA (depending on the antibody) in 1x TBS-T (20 mM Tris-HCl pH 7.4, 150 nM NaCl and 0.1% Tween-20)

for 30 minutes at RT before incubation with primary antibodies overnight at 4°C. After three washings with TBS-T, the incubation with secondary antibodies (horseradish peroxidase (HRP)-conjugated sheep anti-mouse or donkey anti-rabbit IgG, GE Healthcare) was performed for 1 h at RT. The membranes were incubated for 1 minute with WesternBright™ Chemiluminescent Detection Reagent (Advansta) (800 µl H_2O mixed with 200 µl of both reagents) after three washings in TBS-T. The protein signals were detected by Fusion Solo (Vilber Lourmat).
The following antibodies were used: BRCA1 (Rabbit, dilution 1:500, Santa Cruz sc6954); Lamin B1 (Rabbit, dilution 1:1000, Abcam ab16048); MYH (Mouse, 1:333, Abcam ab55551); OGG1 (Rabbit, dilution 1:500, Abcam ab124741). RAD51 (Rabbit, dilution 1:1000, Santa Cruz sc293); RNaseH2A (Rabbit, 1:1000, GeneTex, GTX85020). HRP-conjugated secondary anti-mouse and anti-rabbit antibodies (GE Healthcare) were used at a dilution of 1:5000.

Pulsed field gel electrophoresis (PFGE)
PFGE was performed as described previously (1). Briefly, cells were transfected with siRNA in 6-well plates, re-seeded into 10 cm dishes 48 hours after transfection and treated the day after with 10 µM olaparib for 24 hours. Cells were then harvested by trypsinization and agarose plugs were generated containing 250’000 cells/plug. Quantifications were performed using ImageJ software, and graphs generated using GraphPad Prism.

SUPPLEMENTARY FIGURE LEGENDS

Figure S1. A, Quantitative RT-PCR analysis of BRCA1, OGG1 and MYH mRNA in the siRNA-treated A2780 cells. The experiment was carried out in triplicate ± s.d. Asterisk indicates the extent of statistical significance, calculated by Two-Way ANOVA test in BRCA1 panel and by *t*-test in OGG1 and MYH panels (*, p<0.05; **, p<0.01; ***, p<0.001; ****, p<0.0001). **B**, Representative Western blot analysis of extracts of the siRNA treated A2780 cells.

Figure S2. A, Depletion of OGG1 attenuates olaparib toxicity in HEK293 cells lacking BRCA1. Significance: siLuc – BRCA1; siBRCA1 – siBRCA1/siOGG1. **B**, Depletion of MYH attenuates olaparib toxicity in HEK293 cells lacking BRCA1. Significance: siLuc – BRCA1; siBRCA1 – siBRCA1/siMYH. The results in A, B are means of at least three independent experiments, each carried out in triplicate ± s.d. Asterisk indicates the extent of statistical significance, calculated by Two-Way ANOVA test (*, p<0.05; **, p<0.01; ***, p<0.001; ****, p<0.0001).

Figure S3. Depletion of RNaseH2A in A2780 cells gives rise to DSBs that are additive with those arising from downregulation of BRCA1. **A**, Western blot of extracts of the A2780 cells treated with the indicated siRNAs. **B**, Representative PFGE profile of pellets of A2780 cells treated with the indicated siRNAs, followed by 10µM olaparib. **C**, Quantification of the image shown in panel B.

Figure S4. Western blot analyses of extracts of cells treated with the indicated siRNAs. **A**, Western blot of A2780 cells treated with the indicated siRNAs and grown under hypoxic (1% O_2) conditions. Activation of hypoxia-inducible factor 1 (HIF1) was included to show that the cells were indeed hypoxic. **B**, Western blot of BRCA1-mutated SUM149PT cells treated with the indicated siRNAs. **C**, Western blot of chromatin extracts of A2780 cells treated with the indicated siRNAs.

Reference

1. Hanada, K., Budzowska, M., Davies, S.L., van Drunen, E., Onizawa, H., Beverloo, H.B., Maas, A., Essers, J., Hickson, I.D. and Kanaar, R. (2007) The structure-specific endonuclease Mus81 contributes to replication restart by generating double-strand DNA breaks. *Nat Struct Mol Biol*, **14**, 1096-1104.

Figure S1

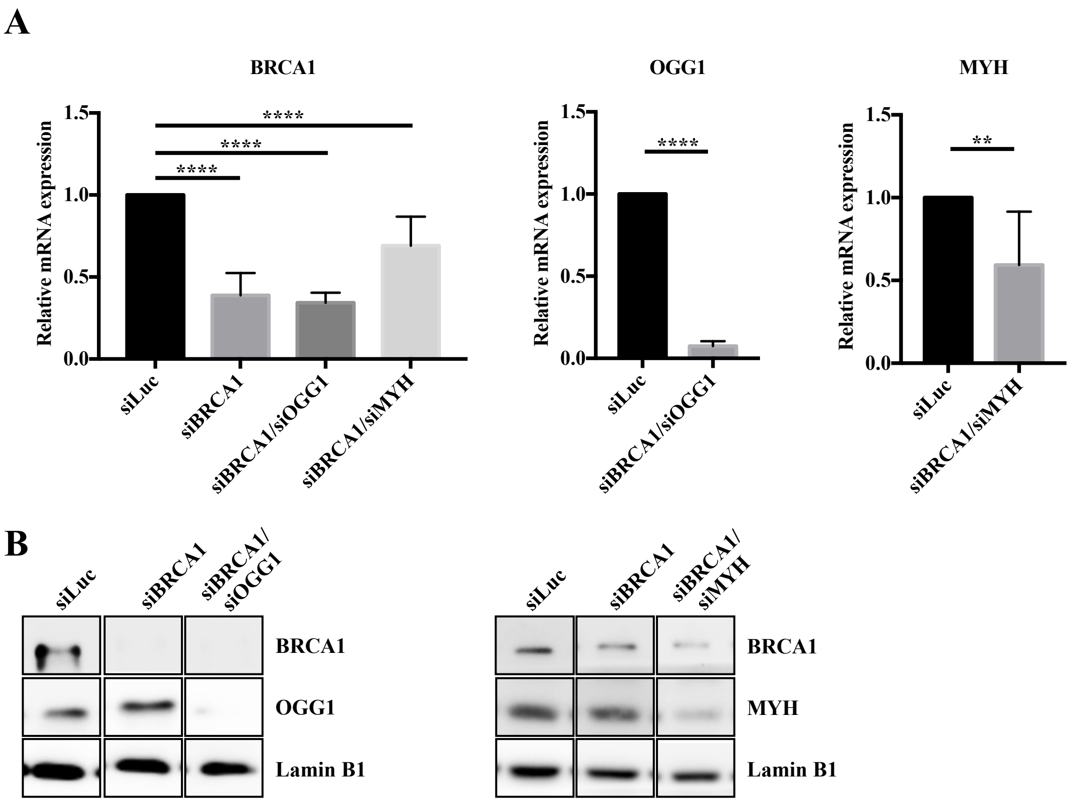

A
BRCA1
OGG1
MYH
Relative mRNA expression
1.5
1.0
0.5
0.0
****
**
siLuc
siBRCA1
siBRCA1/siOGG1
siBRCA1/siMYH
B
siBRCA1/
siOGG1
siMYH
BRCA1
OGG1
MYH
Lamin B1

Figure S2

A

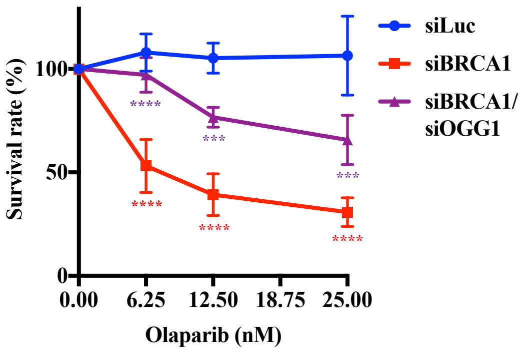

Survival rate (%)
100
50
0
0.00
6.25
12.50
18.75
25.00
Olaparib (nM)
siLuc
siBRCA1
siBRCA1/
siOGG1
****
***
***
****
****
****

B

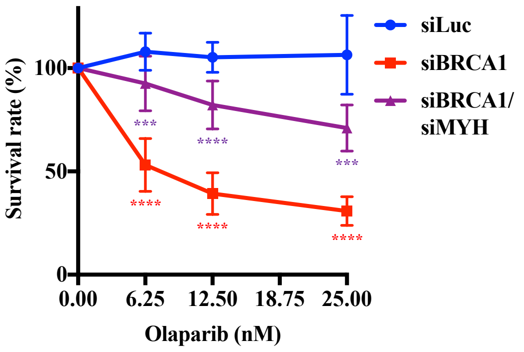

Survival rate (%)
100
50
0
0.00
6.25
12.50
18.75
25.00
Olaparib (nM)
siLuc
siBRCA1
siBRCA1/
siMYH
***
****
***
****
****
****

Figure S3

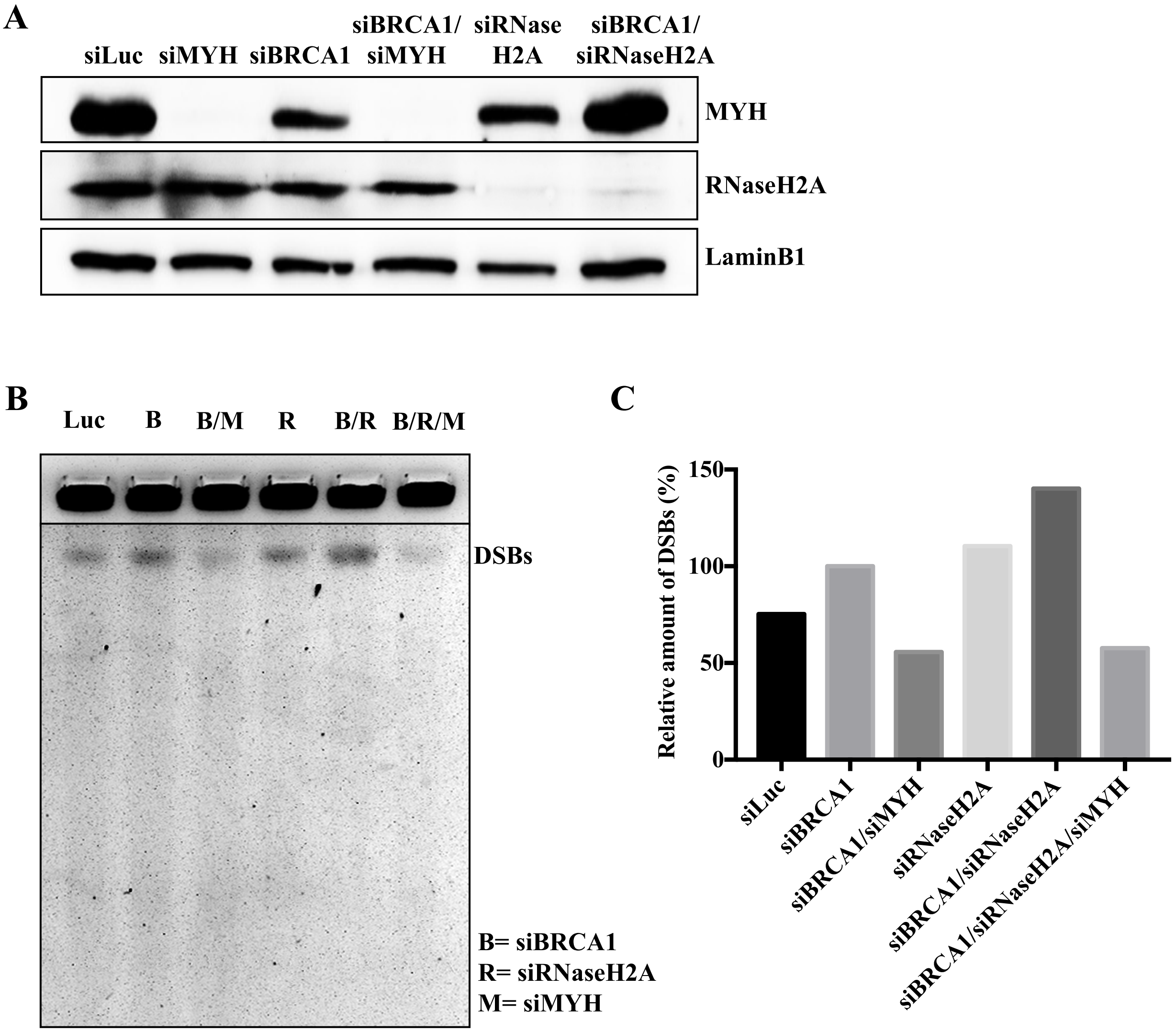
A
siLuc
siMYH
siBRCA1
siBRCA1/
siMYH
siRNase
H2A
siBRCA1/
siRNaseH2A
MYH
RNaseH2A
LaminB1
B
Luc
B
B/M
R
B/R
B/R/M
DSBs
B= siBRCA1
R= siRNaseH2A
M= siMYH
C
Relative amount of DSBs (%)
150
100
50
0
siLuc
siBRCA1
siBRCA1/siMYH
siRNaseH2A
siBRCA1/siRNaseH2A
siBRCA1/siRNaseH2A/siMYH

Figure S4

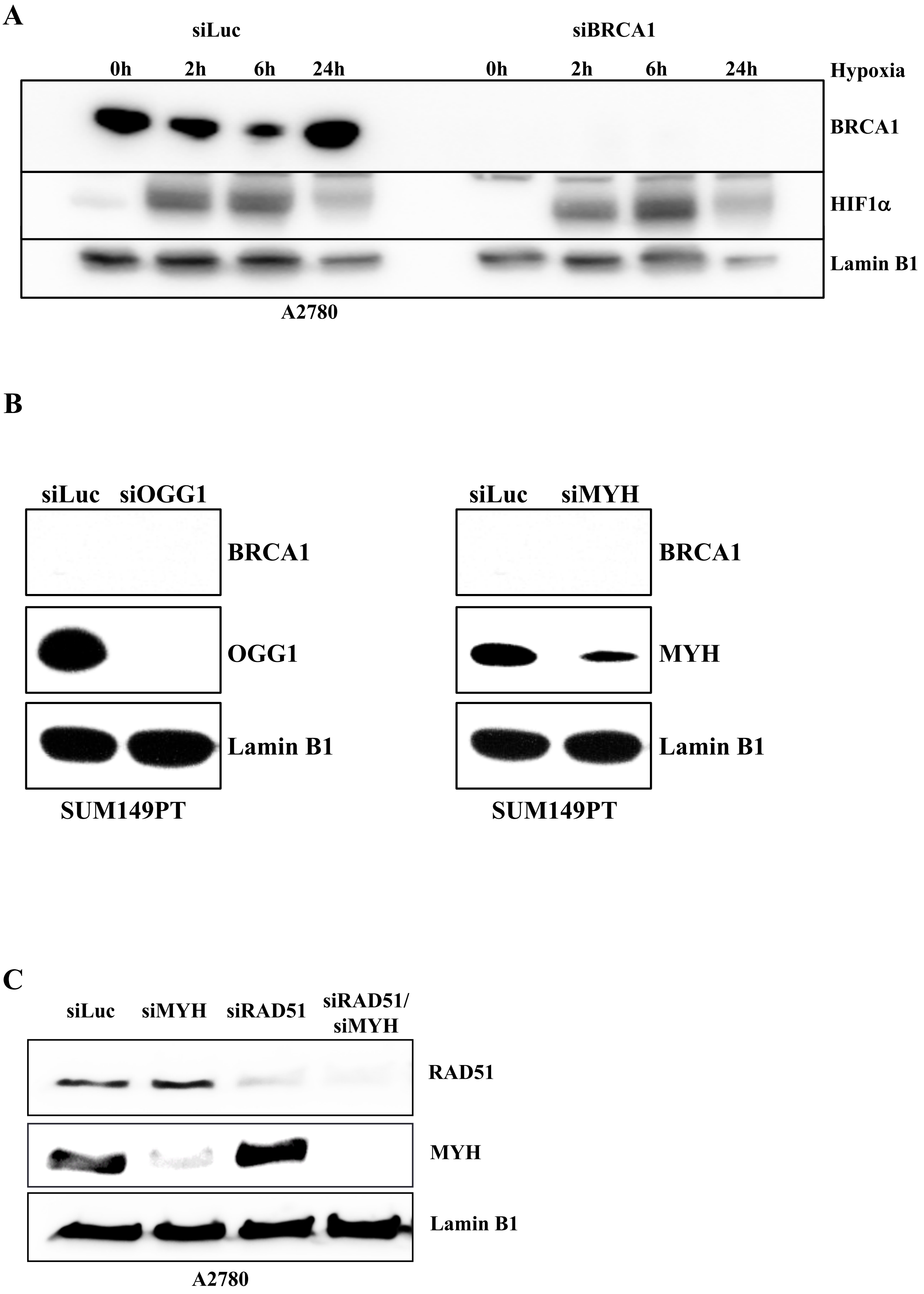
A
siLuc
siBRCA1
0h
2h
6h
24h
0h
2h
6h
24h
Hypoxia
BRCA1
HIF1α
Lamin B1
A2780
B
siLuc
siOGG1
BRCA1
OGG1
Lamin B1
SUM149PT
siLuc
siMYH
BRCA1
MYH
Lamin B1
SUM149PT
C
siLuc
siMYH
siRAD51
siRAD51/
siMYH
RAD51
MYH
Lamin B1
A2780
